# Shifting the optimal stiffness for cell migration

**DOI:** 10.1038/ncomms15313

**Published:** 2017-05-22

**Authors:** Benjamin L. Bangasser, Ghaidan A. Shamsan, Clarence E. Chan, Kwaku N. Opoku, Erkan Tüzel, Benjamin W. Schlichtmann, Jesse A. Kasim, Benjamin J. Fuller, Brannon R. McCullough, Steven S. Rosenfeld, David J. Odde

**Affiliations:** 1 Department of Biomedical Engineering, University of Minnesota, 312 Church Street SE, Minneapolis, Minnesota 55455, USA; 2 Brain Tumor and Neuro-Oncology Center, Cleveland Clinic, 9500 Euclid Avenue, Cleveland, Ohio 44195, USA; 3

## Abstract

Cell migration, which is central to many biological processes including wound healing and cancer progression, is sensitive to environmental stiffness, and many cell types exhibit a stiffness optimum, at which migration is maximal. Here we present a cell migration simulator that predicts a stiffness optimum that can be shifted by altering the number of active molecular motors and clutches. This prediction is verified experimentally by comparing cell traction and F-actin retrograde flow for two cell types with differing amounts of active motors and clutches: embryonic chick forebrain neurons (ECFNs; optimum ∼1 kPa) and U251 glioma cells (optimum ∼100 kPa). In addition, the model predicts, and experiments confirm, that the stiffness optimum of U251 glioma cell migration, morphology and F-actin retrograde flow rate can be shifted to lower stiffness by simultaneous drug inhibition of myosin II motors and integrin-mediated adhesions.

## Abstract (web summary)

Cell migration is sensitive to environmental stiffness, but how cells sense optimal stiffness is not known. Here the authors develop a model that predicts that the optimum can be shifted by altering the number of active molecular motors and clutches, and verify their model in two cell types.

Several models have been proposed to describe optimality in cell migration, beginning with the adhesion strength model of cell migration of DiMilla *et al*.^1^. This model provided a theoretical framework for understanding the experimental observation that cells migrate fastest at intermediate adhesion strength^2^. However, it did not consider migration in compliant environments that is presumably the norm for migration *in vivo*. In addition, cell migration *in vitro* is highly sensitive to the mechanical stiffness of the environment, but the theoretical basis for these effects remains to be established. Since the observation of stiffness-sensitive cell migration by Lo *et al*.^3^, other cell migration models have provided some basis for stiffness sensitive cell migration by incorporating stiffness-dependent functions for force transmission^4^,^5^. However, such approaches introduce stiffness dependence as an assumption a priori, as opposed to having it naturally emerge as an output of the model. Therefore, we currently lack a theoretical explanation for how cell migration depends on the mechanochemical environment and how cells determine the optimal stiffness of their environment.

We and others have previously presented a model of cellular force transmission based on the motor–clutch hypothesis^6^ that naturally predicts biphasic stiffness dependence as an outcome of the model^7^,^8^,^9^. In this model, intracellular molecular motors, such as myosin II, transmit force to the external environment through rigid actin filament bundles and compliant transmembrane molecular ‘clutches', such as integrins (Fig. 1a). In addition, the motor–clutch-based modelling was used to study the effect of substrate stress relaxation on cell spreading, collective cell durotaxis and *ex vivo* cell migration as a function of adhesion molecule expression^10^,^11^,^12^. Our messenger RNA (mRNA) analysis of U251 human glioma cells has identified the most likely candidates for components of the motor–clutch model based on a previously published list^13^ of cell migration genes (Supplementary Table 1). We previously performed a detailed sensitivity analysis on this model and determined that dual parameter changes were needed to account for the broad range of stiffness optima seen experimentally^14^. Specifically, coordinately increasing the expression of molecular motors and clutches shifted the stiffness optimum for maximal force transmission to higher stiffness^14^,^15^. However, our previous study only modelled a single F-actin-based protrusion, and therefore it is not clear whether the optimum shifting predicted by the motor–clutch model would occur in a simulation of an entire cell. Also, the motor–clutch model does not directly predict cellular level features, including cell area, shape and migration, the last being functionally important in nervous system development, immune response and cancer progression.

To address these issues, we developed a stochastic whole cell migration simulator built from the motor–clutch model to simulate cell migration in compliant microenvironments while enforcing force and mass balances. The model predicts a stiffness optimum that can be shifted by altering the number of active molecular motors and clutches. We then experimentally tested the model predictions under conditions of varying mechanical stiffness and coordinate shifts in the activity of motors and clutches. Our results confirmed the model predictions and illustrated the ability to shift the optimal stiffness by altering the number of motors and clutches.

## Results

### Cell migration simulator

**Figure 1:**
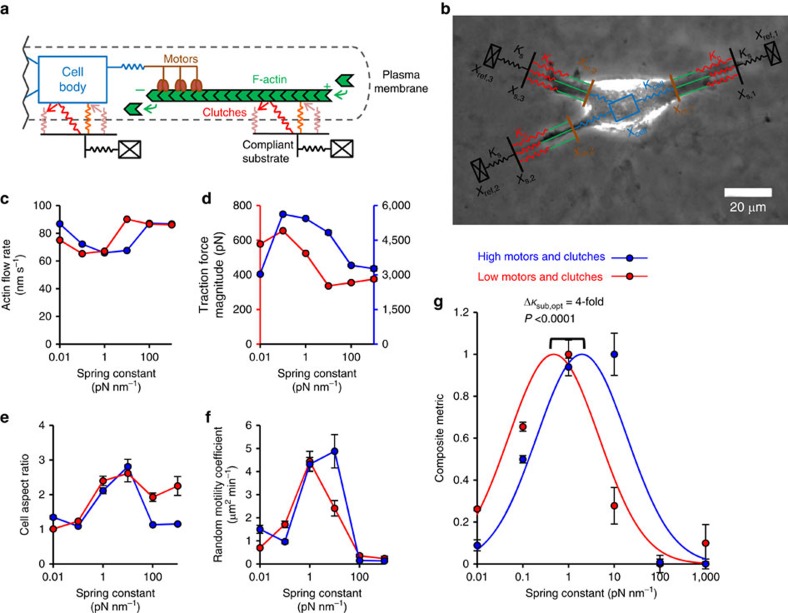
Cell migration simulator. (**a**) Schematic of a motor–clutch module attached to the central cell body. Additional modules may also extend from the cell body but are not shown here for simplicity. (**b**) Representative schematic of the cell migration simulator overlaid on top of a phase-contrast image of U251 glioma cell. This image demonstrates how the simulator captures the three main protrusions of the cell. (**c**–**f**) Plots of simulator outputs for the cases of low (1,000 motors and 750 clutches) and high (10,000 motors and 7,500 clutches) are shown. (**c**) For the low case, the actin retrograde flow minimum occurs around a spring constant of 0.1 pN nm^−1^, and for the high case it occurs at ∼1 pN nm^−1^. (**d**) For both the low and high number of motor and clutches cases, the traction force maximum occurs at ∼0.1 pN nm^−1^ and the high case producing ∼10-fold more force. (**e**) For both low and high motors and clutches, cell aspect ratio has a maximum of ∼10 pN nm^−1^. (**f**) For low motors and clutches, random motility coefficient peaks at ∼10 pN nm^−1^, whereas for high motors and clutches, it peaks at ∼1 pN nm^−1^. (**g**) The composite metric, created to mathematically combine all four metrics, and fit Gaussian curves' peaks show a statistically significant shift in the optimum stiffness for the low and high motors and clutches cases (*P*=0.0001). All error bars are s.e.m. The number of observations for each condition can be found in Supplementary Table 3.

While the motor–clutch model set a foundation for migration sensitivity to stiffness, it did not describe the direct link between force transmission sensitivity and cell migration sensitivity. To investigate this link, we developed a cell migration simulator based on our earlier motor–clutch model. In this simulator, we linked together multiple motor–clutch systems, termed ‘modules', such that the modules each exert a force on a central cell body (Fig. 1a,b, see cell migration simulator parameters values in Supplementary Table 2). The resulting cell migration arises due to a force balance among the motor–clutch modules and the cell body as motors build load and clutch bonds to the substrate break stochastically, thus releasing load.

Our previous theoretical analysis of a single motor–clutch predicted that the optimal stiffness can be readily shifted by coordinately changing the numbers of motors and clutches^14^,^15^. To test whether simulated cells exhibit an optimal stiffness for cell migration, and whether such a shift in the optimal stiffness occurs in our whole cell migration simulator, we simulated cell migration at high and low motor and clutch numbers. The cell migration simulator predicted a stiffness optimum for F-actin retrograde flow rate that was shifted by altering the numbers of motors and clutches (Fig. 1c), consistent with the results for the simpler motor–clutch model that only modelled one protrusion^14^. The simulator also predicted a stiffness optimum for cell traction force that was reduced by altering the numbers of motors and clutches (Fig. 1d). However, the maximum of the simulated cell aspect ratio did not show an obvious shift when changing the number of motors and clutches (Fig. 1e). Finally, the cell migration simulations exhibited a stiffness optimum that was shifted to a higher stiffness by increasing the numbers of motors and clutches (Fig. 1f)—again consistent with our previous motor–clutch analysis^14^ (simulation movies depicting cell shape and migration are provided for 0.1, 10 and 1,000 pN nm^−1^ substrates for low number of motors and clutches; Supplementary Movie 1). A composite metric was created to mathematically combine all four metrics that also shows an increase in the stiffness optimum caused by coordinately increasing the number of motors and clutches in the cell migration simulator (Fig. 1g).

### U251 glioma cell migration and morphology

**Figure 2:**
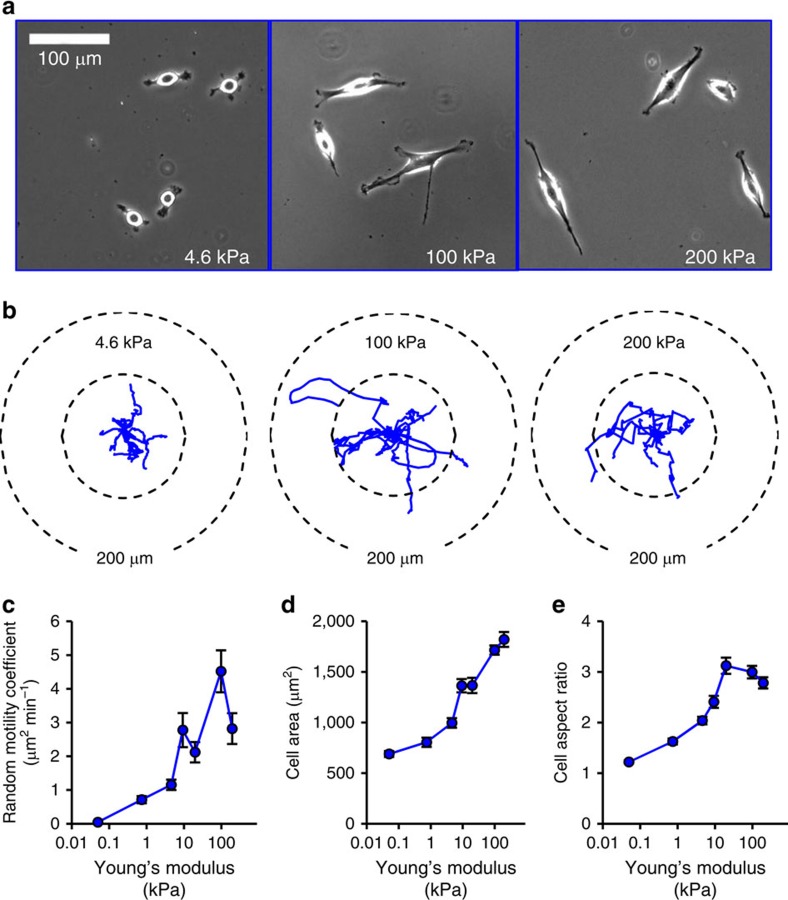
U251 glioma cell migration and morphology have maxima with respect to substrate Young's modulus. (**a**) Representative images of U251 glioma cells on 4.6, 100 and 200 kPa Young's moduli polyacrylamide gels. (**b**) Wind-rose plots of cell trajectories on 4.6, 100 and 200 kPa. Ten randomly selected cell trajectories over 10 h are shown for each condition. (**c**) Cell random motility coefficient has a maximum between 20 and 200 kPa (*P*=0.04). (**d**) Projected cell area has a potential maximum at ∼200 kPa or higher. (**e**) Cell aspect ratio has a potential maximum at ∼9 kPa. All error bars are s.e.m. The number of observations for each experiment can be found in Supplementary Table 3.

To experimentally test the predictions of the cell migration simulator, U251 glioma cells were cultured on polyacrylamide hydrogel (PAG) substrates of varying Young's modulus and surface-immobilized collagen type I (formulations in Supplementary Fig. 1). Based on our cell migration simulator, cell migration rate and morphology are predicted to be substrate stiffness sensitive. As shown in the representative images in Fig. 2a, cell morphology depended on the substrate stiffness, such that on a 4.6 kPa gels the cells were small and round, while on a 100 kPa gel they were larger and more elongated.

To quantify cell migration and morphology, an image segmentation algorithm was used to estimate cell position, spread area and aspect ratio (Supplementary Fig. 2). Figure 2b shows representative wind-rose plots of cell trajectories on 4.6, 100 and 200 kPa, demonstrating the difference in cell migration. Each plot shows the trajectories of 10 randomly selected cells over 10 h. Qualitatively, the cells on 100 kPa migrate further in 10 h than the cells on either 4.6 or 200 kPa. Cell positions were used to calculate the mean squared displacement for each cell over time intervals ranging from 15 min to 5 h (Supplementary Fig. 2e) to estimate cell random motility coefficients. Over a stiffness range of 50 Pa to 200 kPa, the mean cell random motility coefficient had a maximum at ∼100 kPa (Fig. 2c). Similarly, mean cell area increased with increasing stiffness, although it did not exhibit a clear maximum (Fig. 2d), a feature that we revisit later in the drug treatment studies. Similar to migration rate, the cell aspect ratio exhibited a maximum of ∼20–100 kPa (Fig. 2e). Sample movies of U251 glioma cells on 4.6, 100 and 200 kPa are presented in Supplementary Movie 2. In addition, mRNA expression was quantified for the cells on different stiffnesses to show that the migration differences were not the effect of changes in genes expression on the different stiffnesses. Few cell migration genes showed any significant expression difference among the stiffnesses, and nearly all the expression differences were found between plastic and the PAGs, rather than among the PAGs (Supplementary Fig. 3).

### F-actin retrograde flow and traction strain energy

**Figure 3:**
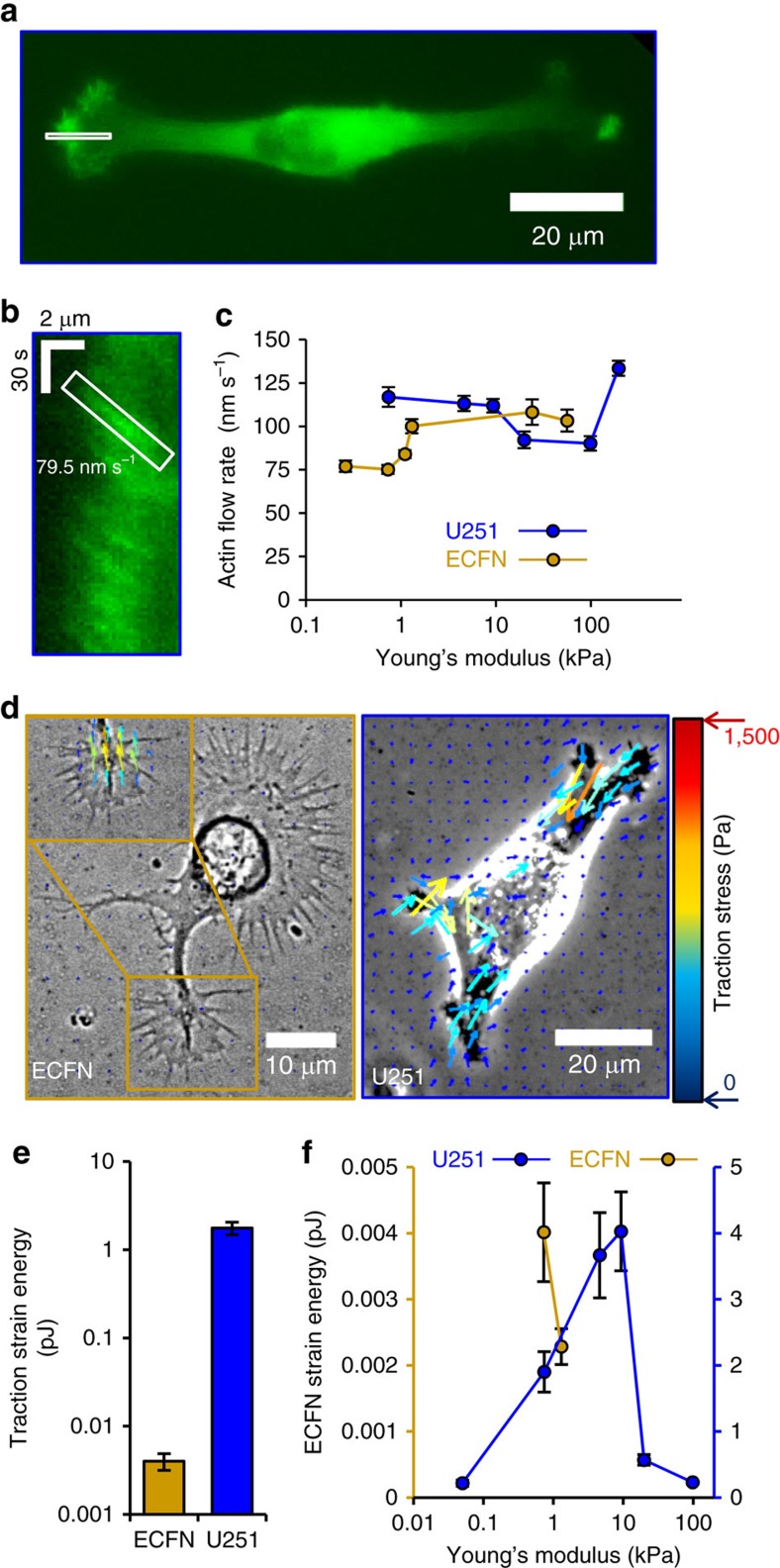
Actin retrograde flow and traction strain energy of U251 glioma cells versus ECFNs. (**a**) Representative fluorescent image of an EGFP-actin U251 glioma cell on a 100 kPa polyacrylamide gel. The boxed region represents an area selected for an actin flow kymograph. (**b**) Actin flow kymograph for a U251 glioma cell on a 100 kPa polyacrylamide gel. Horizontal bar is 2 μm. Vertical bar is 30 s. (**c**) Embryonic chick forebrain neuron (ECFN; data taken from Chan and Odde^7^) and U251 glioma cell actin retrograde flow versus substrate stiffness. U251 actin flow has a minimum between 9 and 200 kPa (*P*=0.004). (**d**) Representative phase-contrast images with traction field overlays of an ECFN and a U251 glioma cell on 700 Pa polyacrylamide gels. Traction vectors were thinned fourfold for ease of visualization. (Inset) Region of ECFN with traction vectors expanded by 60-fold. (**e**) Mean strain energy of ECFNs and U251 glioma cells on 700 kPa polyacrylamide gels. U251 strain energy is significantly higher than for ECFNs (*P*=10–24). (**f**) U251 stain energy has a maximum between 4.6 and 20 kPa (*P*=0.0002). ECFN data were adapted from Chan and Odde^7^. All error bars are s.e.m. The number of observations for each experiment can be found in Supplementary Table 3.

Previous experimental measurements of embryonic chick forebrain neuron (ECFN) behaviour suggests that they have minimal actin flow and maximal traction strain energy on a stiffness of ∼1 kPa^7^,^14^. Because ECFNs have a lower optimum stiffness than the U251 glioma cells (∼1 kPa for ECFNs versus ∼100 kPa for U251 glioma cells), then, based on the cell migration simulator predictions (Fig. 1c), this suggests the possibility that ECFNs have fewer motors and clutches than U251 glioma cells. To experimentally test this prediction, we measured F-actin retrograde flow in EGFP-actin U251 glioma cells cultured on varying stiffness substrates. Based on the cell migration simulator, we expect a shift up in retrograde flow minimum in U251 cells compared with ECFNs. Expression of EGFP-actin has previously been shown not to affect F-actin retrograde flow rate in neurons, a finding that we now also confirm for U251 glioma cells in Supplementary Fig. 4a–c. Figure 3a shows a representative fluorescent image of an EGFP-actin U251 cell, and Fig. 3b shows a representative kymograph of actin flow at the edge of a U251 glioma cell. According to the cell migration simulator (Fig. 1c), the increase in optimal stiffness from ∼1 kPa for neurons to ∼100 kPa for glioma cells should correspond to an ∼100-fold increase in the minimum in F-actin retrograde flow rate. As shown in Fig. 3c, the actin flow for U251 cells has a minimum between 20 and 100 kPa that occurs at the Young's modulus approximately two orders of magnitude greater than the minimum for ECFNs as predicted for ∼100-fold increase in the number of motors and clutches.

If the higher optimal stiffness for glioma cells versus neurons is due to an ∼100-fold increase in the numbers of motor and clutches, then the cell migration simulator predicts that cell traction forces should be two orders of magnitude larger for U251 cells than for ECFNs. Alternatively, sensitivity analysis of the motor–clutch showed that the optimal stiffness can be increased by orders of magnitude via a coordinate increase in the on and off rate constants for the clutches^14^ that would predict no change in traction force between U251 cells and ECFNs. Using Fourier transform traction cytometry^16^, we estimated traction strain energy for both cell types on the same stiffness. As shown in Fig. 3d, the U251 glioma cells clearly transmit more force, and when plotted on the same scale, the traction vectors for ECFNs are barely detectable compared with those for U251 glioma cells. Consistent with the prediction of increased motors and clutches, and inconsistent with the prediction of increased on–off clutch kinetics, the mean U251 glioma cell traction strain energy on 700 Pa modulus gels is ∼400 times greater than the mean ECFN traction strain energy (Fig. 3e; measurements validated against measurement noise in Supplementary Fig. 4d,e). The greater force transmission for U251 glioma cells suggests that they express at least two orders of magnitude more motors and clutches than the ECFNs. We have previously shown that clutches must approximately balance motors to maintain stiffness sensitivity^14^. Therefore, since actin flow is stiffness sensitive for both cell types, the expression of clutches also likely differs by at least two orders of magnitude. Finally, if the numbers of motors and clutches is increasing, then the cell migration simulator predicts an optimal stiffness for maximal force transmission that will be shifted to higher stiffness for glioma cells than neurons. As predicted for increased motors and clutches, the mean traction strain energy for U251 glioma cells has a maximum of ∼10 kPa, an order of magnitude higher than the potential maximum of ECFN strain energy that is ∼1 kPa (Fig. 3f; technical details for high stiffness measurements described in Supplementary Fig. 4e,f). Together, the experimental measurements of F-actin retrograde flow rate and cell traction force support a motor–clutch-based model where U251 glioma cells express ∼100-fold greater motors and clutches than ECFNs, and argue against a model where clutch on–off kinetics are altered. As a result, we conclude that the large difference in stiffness optima between glioma cells and neurons is mainly due to glioma cells expressing ∼100-fold greater numbers of motors and clutches than neurons.

### Simultaneous inhibition of motors and clutches

As shown in Fig. 1g, the cell migration simulator predicts a decrease in the optimal stiffness upon coordinate decrease in both motors and clutches. To further test experimentally the prediction that coordinate changes in the numbers of active motors and clutches shift the optimum stiffness for a cell, we partially inhibited myosin II motors via blebbistatin and partially inhibited integrin-mediated adhesions to the collagen-coated substrate via cyclo(RGDfV) competitive binding using the U251 glioma cells.

**Figure 4:**
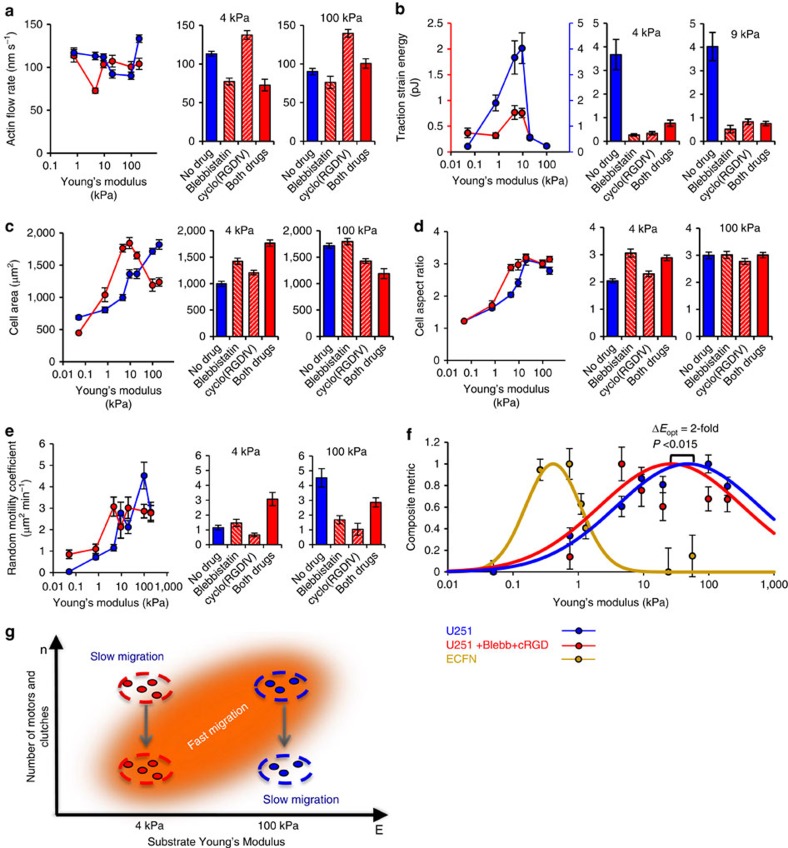
Simultaneous inhibition of motors and clutches shifts the optimum stiffness to lower Young's modulus. (**a**) Addition of both drugs shifted the minimum actin flow rate to ∼4.6 kPa. cylco(RGDfV) alone increased actin flow on both 4.6 and 100 kPa, while blebbistatin alone reduced actin flow on 4.6 kPa but not significantly on 100 kPa. The combined drug treatment reduced actin flow on 4.6 kPa but did not significantly affect actin flow on 100 kPa. (**b**) Addition of both drugs decreased traction strain energy by approximately fourfold on all stiffnesses. The maximum traction strain energy maintained its maximum at ∼9 kPa. However, the combined drug treatment increased the traction strain energy compared with either individual drug treatment on 4.6 kPa. (**c**) Addition of both drugs caused the maximum area to be ∼9 kPa. On 4.6 kPa, the addition of both drugs increased the cell area, while on 100 kPa they decreased the cell area. (**d**) Addition of both drugs did not shift the maximum aspect ratio. On 4.6 kPa, blebbistatin individually and both drugs combined increase the aspect ratio. None of the drugged cases had a significant effect on the aspect ratio on 100 kPa. (**e**) Simultaneous addition of 6 μM blebbistatin and 0.6 μM cyclo(RGDfV) shifts the potential maximum U251 glioma cell random motility coefficient to ∼4.6 kPa. On 4.6 kPa, addition of both drugs increases the random motility coefficient compared with the no drug, blebbistatin and cylco(RGDfV) cases. On 100 kPa, all three drugged cases are lower than the no drug case, but addition of both drugs increases the random motility coefficient compared with either single drug case. (**f**) A composite metric with corresponding Gaussian fit curves demonstrates the shifting in stiffness optimum among U251 glioma cells, U251 glioma cells treated with blebbistatin and cyclo(RGDfV) and ECFNs. Gaussian fit curves peaks show a statistically significant shift in the optimum stiffness for the untreated and treated U251 glioma cell composite metric (*P*=0.015). (**g**) Illustration of how same drug treatment may result in opposite effects in different mechanical environments. *P* values for all bar chart comparisons are presented in Supplementary Table 4. All error bars are s.e.m. The number of observations for each experiment can be found in Supplementary Table 3.

As predicted by the cell migration simulator, the combined drug treatment shifted the minimum of actin flow to around 4.6 kPa (Fig. 4a). This occurred due to a decrease in the actin flow rate on 4.6 kPa, but the actin flow rate on 100 kPa was not significantly affected. The maximum in traction stain energy did not shift out of the control range due to the combined treatment, but the traction strain energy was reduced as shown in Fig. 4b. On 4.6 kPa, each drug individually decreased the traction strain energy by about an order of magnitude, but the combined drug treatment actually increased the traction strain energy compared with the single drug cases. This indicates that the addition of a traction-inhibiting drug can actually *increase* the traction strain energy in the presence of the other traction-inhibiting drug, an effect predicted by the model. On 9.3 kPa, the traction strain energy was reduced by about an order of magnitude in each single drug case and the combined treatment.

The combined drug treatment showed an optimum for cell area at ∼10 kPa, implying that untreated cells' optimum is ∼200 kPa or higher (Fig. 4c). The combined treatment had opposite effects on 4.6 kPa where it increased cell area and on 100 kPa where it decreased cell area. Furthermore, combined drug treatment did not shift the optimum for cell aspect ratio (Fig. 4d). On 4.6 kPa, this treatment increased the cell aspect ratio, but it had no significant effect on aspect ratio on 100 kPa. Finally, Fig. 4e shows a potential shift in the maximum of the random motility coefficient. More importantly, the random motility coefficient on 4.6 kPa increased in the combined drug case compared with the cases with no drug or each drug individually. Conversely, the combined drug treatment decreased random motility compared with the no drug case on 100 kPa. Interestingly, the individual drug treatments each decreased the motility more than the combined treatment on 100 kPa, indicating that the addition of a motility-inhibiting drug partially rescues motility in the presence of the other motility-inhibiting drug.

The combined data of random motility coefficient, cell area, cell aspect ratio, actin flow rate and traction strain energy indicate that the coordinate inhibition of motors and clutches reduced the cell optimum stiffness.

Furthermore, Fig. 4f demonstrates this fact by presenting a metric that combines all five types of data for U251 glioma cells with no drug and with the combined drug treatment. These results are also compared with the composite metric for ECFN actin flow and traction strain energy. The ECFNs are expected to have low numbers of motors and clutches, and correspondingly they have a low optimum stiffness near 1 kPa. The U251 glioma cells have a higher optimum stiffness near 50 kPa. The combination drug-treated U251 glioma cells, which are expected to have about an order of magnitude fewer active motors and clutches, in turn have an optimum near 20 kPa. Finally, the number of experimental observations and significance values for comparisons in Fig. 4 are presented in Supplementary Tables 3 and 4.

## Discussion

Here we confirmed a mechanism by which cells are able to determine the optimal stiffness of their environment that was predicted by the cell migration simulator. In addition, the cell migration simulator presented in this work provided accurate and unexpected, quantitatively accurate predictions that were validated experimentally. The values for random motility coefficient, cell aspect ratio and actin flow rate differed at most by ∼25% from the experimental values. This accuracy for each of the three separate metrics of cell behaviour is especially remarkable given that many of the parameter values were order of magnitude estimates taken from broad experimental ranges. The cell migration simulator could also be used in the future to simulate a variety of cell migration behaviours in heterogeneous environments including durotaxis^3^ and confined migration^17^. Although the current version of the simulator acts in two spatial dimensions, it can be extended to three spatial dimensions by allowing modules to extend along a third coordinate and solving a force balance in the third dimension. Perhaps most importantly, the cell migration simulator could be used as an *in silico* diagnostic and treatment tool for diseases such as glioblastoma for which aberrant cell migration is a major contributor to poor prognoses^18^ (Fig. 4g).

## Methods

### Migration simulator description

The cell migration simulator is based on the motor–clutch model of cellular force transmission^7^. In this simulator, the cell is composed of *j* motor–clutch modules, each following rules as previously described^7^,^14^. Briefly, n_c,j_ clutches may bind from F-actin within the cell to a compliant substrate outside of the cell at rate *k*_on_. These clutches unbind at a rate, *k*_off,i_, that depends on the clutch force, *F*_c,i_, according to the Bell model^19^ in equation (1) where k^*^_off_ is the unloaded clutch off-rate and *F*_b_ is the characteristic bond rupture force.


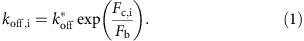


The clutches and the compliant substrate are each modelled as Hookean springs with spring constants *κ*_c_ and *κ*_*s*_ respectively. The rigid F-actin is retracted by *n*_m,j_ molecular motors, each with stall force *F*_m_ and unloaded velocity v^*^_m_, at rate v_m,j_ as described by equation (2) in which *x*_s,j_ is the substrate displacement for the *j*^th^ module.


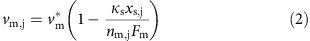


Additional rules were added to govern F-actin polymerization and depolymerization in the module. The polymerization speed, *v*_p_, was defined to be proportional to the ratio of G-actin, A_G_, to the total amount of actin in the cell, *A*_T_, as in equation (3) where v^*^_p_ is the maximum F-actin polymerization speed.


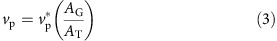


F-actin was depolymerized when it passed the motor position for its module. Clutches were also forced to unbind if their F-actin attachment point passed the motor position. Each module contained a cell spring with spring constant *κ*_cell_ that connected the module motors to the central cell body. The forces along each module balance according to equation (4) where *x*_cell,j_ is the extension of the cell spring for the *j*^th^ module, and *x*_c,i_ is the extension of the *i*^th^ clutch of the module.


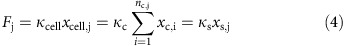


There were also *n*_c,cell_ clutches associated with the central cell body that balance a central substrate displacement as in equation (5) describing the force on the cell body, *F*_cell_.


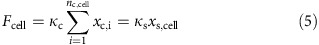


An overall cell force balance was imposed such that the cell force and all module forces summed to zero as in equation (6) where *n*_mod_ is the number of modules.


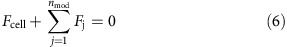


Modules of initial length *l*_in_ were created at rate *k*_mod_ that was proportional to the ratio of G-actin to the total actin raised to the fourth power^20^,^21^ as in equation (7) where k^*^_mod_ is the maximum rate of module formation.


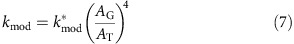


When a module was created, it was assigned motors and clutches from a pool of free motors, *n*_m,free_, and a pool of free clutches, *n*_c,free_, according to equations (8) and (9) where *n*_m,tot_ and *n*_c,tot_ are the total numbers of motors and clutches in the cell, and n^*^_m_ and n^*^_c_ are the maximum numbers of motors and clutches for a module.


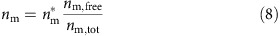



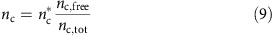


The direction of the module was assigned randomly in the two dimensional plane. Modules were capped at rate *k*_cap_, eliminating further polymerization for the capped modules, and they were destroyed if their length was less than a minimum length, *l*_min_. Upon module destruction, all motors, clutches and remaining actin were returned to their respective pools.

### Simulator implementation

The model was implemented using a direct Gillespie Stochastic Simulation Algorithm (SSA)^22^ in Matlab (MathWorks). The time to the next event, t_event_, was calculated by equation (10) where *URN*_1_ is a uniformly distributed random number, and *k*_i_ is the rate of the *i*^th^ of *n* possible events including clutch binding, clutch unbinding, module creation and module capping.


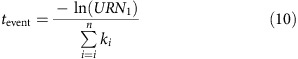


The event *i* to execute was then determined using a second random number, *URN*_2_, such that equation (11) was satisfied.


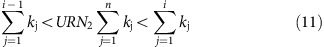


This direct method is computationally efficient because only two random numbers are generated regardless of the number of possible events.

The order of the model algorithm is described below.

1. Initialize a cell with three equally spaced modules of length *l*_in_ and no clutches bound.
2. Calculate all clutch unbinding rates and module birth rate.
3. Calculate time to next event.
4. Determine which event to execute.
5. Calculate F-actin retrograde flow rate for each module.
6. Shorten each module by the product of the F-actin flow rate and the event time.
7. Advance the clutch, substrate and reference positions for each uncapped module by the F-actin polymerization length for the event time.
8. Execute event.
9. Destroy modules shorter than length *l*_min_.
10. Evaluate force balance to determine the new cell body position.
11. Use the force on each module to determine the cell spring extension, clutch extensions and the substrate spring extension.
12. Return to step 2.

### Simulation analysis

The random motility coefficient (*μ*) for simulated two-dimensional cell migration was defined similarly to a diffusion coefficient as in equation (12)^23^.


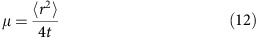


In this equation, 〈*r*^2^〉 is the mean squared displacement of the cell centre in a given time interval, *t*. From this relation, *μ* can be calculated from the slope of 〈*r*^2^〉 versus *t*. This plot was generated for each simulated cell according to the overlap method of mean squared displacement calculation^24^. In this method, all displacements at all possible time intervals are used to generate the plot. To correspond to our experimental cell migration experiments, simulation data at 15 min intervals was used to calculate mean squared displacements, and the first hour of each simulation were excluded from analysis to allow the system to reach steady state (Supplementary Fig. 5). In addition, large displacements in the simulated cell centre of 1 μm s^−1^ were removed. A linear trend line intersecting the origin and inversely weighted by the uncertainty in each data point was fit to the first half of the 〈*r*^2^〉 versus *t* data for each simulated cell. The random motility coefficient was calculated from the slope of the trend line and averaged across all simulated cells at a given condition to obtain the mean random motility coefficient for that condition.

Simulated cell morphology was determined by creating a geometric shape based on the position outputs of the simulation. First, a 10 μm radius circle was centred at the cell body position. Then, 10 μm was added to the length of each module to account for the module starting at the edge of the cell body rather than the central point. Tangent lines to the cell circle were then drawn to connect the module tips to the central circle. The resulting shape was then analysed for aspect ratio using MATLAB ‘regionprops' function. Simulation movies depicting cell shape and migration are provided for 0.1, 10 and 1,000 pN nm^−1^ substrates (Supplementary Movie 1).

Finally, the F-actin flow rate was calculated by taking the mean of the F-actin flow rates for each module, and the traction force was calculated by summing the magnitudes of the force on each module as well as the cell body.

### Preparation of collagen-coated polyacrylamide substrates

Polyacrylamide gel substrates were prepared according to a standard method popularized by Wang and Pelham^25^. Briefly, No. 0 glass bottom culture dishes (MatTek P35G-0-20-C) were treated with 0.1 M NaOH, 97% (3-aminoproyl)trimethoxysilane (Aldrich 281778) and 0.5% glutaraldehyde (Polysciences 01909) to activate the glass surface^26^. Activated culture dishes were stored in a desiccator for up to 2 weeks until use. A prepolymer mixture of 40% acrylamide solution (Fisher BP1402), 2% bis-acrylamide solution (Fisher BP1404), 1 M 4-(2-hydroxyethyl)piperazine-1-ethanesulfonic acid (HEPES, Sigma H6147) solution and deionized water was prepared for the desired Young's modulus (Supplementary Fig. 1). For cell traction experiments, 1% (v/v) 200 nm crimson fluorospheres (Invitrogen F8806) were added to the mixture. After degassing, polymerization was initiated by adding 0.6% (v/v) 1% ammonium persulfate (Bio-Rad 161-0700) solution and 0.4% (v/v)N,N,N′,N′-tetramethylethylenediamine (TEMED, Fisher BP150). Next, 4 μl of polymer solution was quickly pipetted onto the activated glass culture dishes and covered with a 12 mm No. 1.5 circular cover slip (Fisher 12-545-80) to create a gel 40–100 μm thick^7^. After removal of the coverslip, type I rat tail collagen (BD Biosciences 354236) was conjugated to the gel using 0.5 mg ml^−1^ sulfosuccinimidyl 6-(4′-azido-2′-nitrophenylamino) hexanoate (sulfo-SANPAH, Thermo 22589) and 200 μg ml^−1^ collagen solution.

### Characterization of polyacrylamide substrates

The Young's moduli of polyacrylamide gels made from different formulations were measured using a bead indentation technique^7^ based on Hertzian indentation theory^27^,^28^ (Supplementary Fig. 1). Glass beads (Polysciences) with radii ranging from 0.03 to 5 mm were placed on 700–1,000 μm thick gels created by pipetting 300 μl of polymer solution onto an activated glass culture dish and covered with 25 mm No. 1.5 circular cover slip (Fisher 12-545-102) . The bead contact area was measured using the gravity-settled 200 nm crimson fluorospheres as a marker for the gel surface. The bead indentation depth (*δ*) was calculated from the bead radius (*R*) and the contact radius (*r*) according to equation (13).


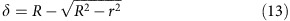


From this indentation depth, the gel Young's modulus (*E*) was calculated using the Poisson ratio of the hydrogel (*ν*), and the buoyancy corrected bead force (*f*) according to equation (14).


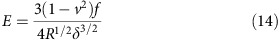


For polyacrylamide gels, *ν*=0.3–0.5 (*ν*=0.3 was used here), and the glass bead density was measured to be ∼2,600 kg m^−3^.

### U251 glioma cell culture

Human glioma cell line U251 was originally obtained from Dr G. Yancey Gillespie (UAB). These cells were authenticated using STR assay (University of Arizona Genetics Core). U251 cells were cultured in vented T25 tissue culture flasks (Becton Dickinson, 353108) for 3–4 days in a 37 °C 5% CO_2_ incubator in media comprising 45.5% F12+GlutaMAX supplement (Gibco 31765-035), 45.5% high glucose Dulbecco's modified Eagle's medium (Gibco 10566-024), 8% heat-inactivated fetal bovine serum (Gibco 10438-026) and 1% penicillin/streptomycin solution (Cellgro 30-001-CI). Cells were removed from the flask using 0.05% trypsin with EDTA in Hanks' balanced salt solution (Gibco 25-052-CI) and transferred to the collagen-coated gels at a density of ∼5 cells per mm^2^. The cultured gels were placed in the 37 °C 5% CO_2_ incubator overnight before imaging the next day.

### Formation of EGFP-actin expressing U251 glioma cell line

EGFP-actin plasmid (provided by Paul Letourneau, University of Minnesota) was transfected into U251 glioma cells using a GenePulser XCell electoroporator (Bio-Rad). Approximately 10^6^ cells in 400 μl of antibiotic-free media were placed in a 4 mm electroporation cuvette (Molecular BioProducts 5540) with 10 μg of plasmid. The cells were electroporated using nine 0.1 s square wave pulses of 480 V at 1 s intervals, achieving 5–10% transfection efficiency. The transfected cells were transferred to collagen-coated gels at a density of ∼5 cells per mm^2^. The cultured gels were placed in the 37 °C 5% CO_2_ incubator overnight before imaging the next day.

### Live cell imaging

Cells were imaged using a Nikon Eclipse TE200 inverted microscope and a CoolSnap HQ2 CCD camera (Photometrics) controlled by MetaMorph v7.1.4 imaging software (Molecular Devices). For all imaging, cell culture dishes were placed in a Bold Line top stage incubator (Okolab) at 37 °C and 5% CO_2_. Cell migration images were taken using phase-contrast imaging and a Plan Fluor 10 × /0.30NA objective. Time-lapse movies were taken at 15 min intervals for 16–24 h. Sample movies of U251 glioma cells on 4.6, 100 and 200 kPa are presented in Supplementary Movie 2. EGFP-actin flow images were taken using a Plan Fluor ELWD 40 × /0.60 NA objective with epifluorescence microscopy illumination provided by a PhotoFluor II light source (89 North) and EGFP filter set. Time lapse movies were taken at 2 s intervals for 3 min. Additional F-actin flow movies were taken with phase-contrast imaging to verify that the expression of EGFP-actin did not alter the actin retrograde flow rate (Supplementary Fig. 4a–c). Sample movies of EGFP-actin flow and phase-contrast actin flow are presented as Supplementary Movies 3 and 4,respectively.

### Cell traction imaging

Cell traction images were taken with a Plan Fluor ELWD 40 × /0.60 NA objective using both phase-contrast imaging and epifluorescence using mCherry/EGFP filter set. A phase-contrast cell image was taken followed by an epifluorescent strained gel image. Media were then removed and 0.05% trypsin with EDTA in Hanks' balanced salt solution (Gibco 25-052-CI) was added to remove cellular adhesions to the substrate, relieving the stress in the gel. At 10 min after adding trypsin, another epifluorescent image was taken of the gel in its relaxed state. For traction measurement on 100 kPa gels, an improved microscope stage insert with better dish stability was used to reduce measurement noise (Supplementary Fig. 4).

### Cell migration analysis

Individual cells were identified in each frame of the cell migration movies by using a custom-written image segmentation algorithm in MATLAB (MathWorks). A Gaussian filter was first applied to remove noise in the images, and cell edges were identified using Sobel edge detection. The identified edges were then dilated to bridge any gaps in the edge detection. Regions enclosed by the edge detection were filled and eroded back to their original size. The user then selected one of the regions (cells) to track throughout the movie. For each frame, the region centroid, area and major and minor axis lengths were recorded (Supplementary Fig. 2a–d). Regions were matched from frame to frame by identifying a region that contained the recorded centroid from the previous frame. If no such region existed, the user manually identified the region corresponding to the cell being tracked, or the user skipped recording for that frame. The algorithm also requested user input if the area of the tracked region increased by >50% or decreased by >25% from frame to frame. In these cases, the user manually confirmed if the region information should be recorded or discarded for that frame. The random motility coefficient (*μ*) for 2D cell migration was calculated using the same method as for the simulated cells by fitting a line to the mean squared displacement versus time plot that is constrained to go through the origin (Supplementary Fig. 2e).

### Actin retrograde flow rate measurement

Actin flow speed was calculated by taking kymographs (space–time plots) along the axis of moving actin features (approximately orthogonal to the local leading edge) near the edges of EGFP-actin U251 cells using the ImageJ multiple kymograph plugin. Actin flow could not be measured on 50 Pa for U251 cells because there were no protrusions long enough to make the measurements. The kymographs were analysed using a custom-written MATLAB (MathWorks) algorithm that cross-correlated each spatial frame along a specified region of the time axis^7^. A Gaussian curve was fit to the average cross-correlation function for the specified region, and the maximum of the curve was taken as the average displacement of actin from frame to frame. This displacement was used in conjunction with the frame rate to calculate the speed.

### Traction strain energy measurement

Cell traction stress fields were measured using a custom-written MATLAB (MathWorks) implementation of Fourier transform traction cytometry^16^. First, background signal was removed from the strained and relaxed gel images using a morphological top hat filter. The phase-contrast image of the cell was then used to create a mask that was overlaid on top of the strained and relaxed gel images. This excluded beads displaced by the cell from the subsequent image registration. The strained and relaxed gel images were roughly registered by two-dimensional Fourier transform cross-correlation and then fully registered to subpixel accuracy by optimizing an affine transformation to maximize the cross-correlation between the two images. In all steps of the registration, the strained image was left unchanged while the relaxed image was transformed. This was done because the strained image and the phase-contrast image were taken at the same time point.

A gel displacement field was calculated by applying particle image velocimetry to the fluorospheres in the strained and relaxed gel images. A coarse displacement field was calculated using a 48 × 48 pixel (7.8 × 7.8 μm) window size with a search neighbourhood extending 100 pixels (16.3 μm) beyond the window. Window displacement was calculated by maximizing the cross-correlation between the strained and relaxed images. A fine displacement field was calculated using a 24 × 24 pixel (3.9 × 3.9 μm) window size with the coarse displacement field as an initial guess for the cross-correlation maximization. Windows for the fine displacement field were overlapped by one half of their length in each dimension for a final lattice spacing of 12 pixels (2.0 μm).

From the displacement field, the traction stress field was calculated using the inverse Boussinesq solution^29^ in Fourier space^16^. Finally, the traction strain energy was calculated by integrating the dot products of the traction vectors (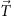) with the displacement vectors (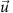) at each position (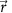) over the area of the image as in equation (15)^16^.


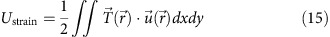


The stain energy calculation provides an advantage over simply summing the magnitudes of the traction field vectors because the dot product with the displacement vectors minimizes the effect of noise in the calculation. Traction vectors that are poorly aligned with their corresponding displacement vectors contribute weakly to the stain energy. However, given the same traction field, the strain energy will decrease with Young's modulus (*E*) because 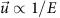, therefore 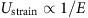. To correct for this effect, the strain energy was multiplied by the substrate Young's modulus to validate that experimental differences in strain energy corresponded to actual differences in cell traction (Supplementary Fig. 4g-i).

### U251 mRNA expression

To collect enough mRNA for expression analysis on different stiffnesses, U251 cells were cultured on large polyacrylamide gels covering the surface of a one well chamber glass slide (Lab-Tek 154453). After 1 day of culture on the gels, mRNA was purified from the cells using an RNeasy Mini Kit (Qiagen 74104). The mRNA samples were then analysed at the University of Minnesota Genomics Center using a HumanHT-12 BeadChip microarray (Illumina BD 103-0204). The mRNA was collected from three U251 glioma cultures each on 4.6, 20 and 200 kPa PAGs as well as plastic for a total of 12 mRNA samples. mRNA counts from the BeadChip were compared with a published list^13^ of cell migration genes to identify the most likely contributors to the motor–clutch model in U251 glioma cells (Supplementary Table 1). The expression levels on the different stiffnesses were compared with each other to identify any significant expression differences among the stiffnesses.

### Composite metrics

For both the simulation and experimental results, a composite metric was created to pool the data of random motility coefficient, cell area, aspect ratio, actin flow rate and traction. To create this composite metric, each data set was scaled such that the minimum value was zero and the maximum value was one. For actin flow rate, the data were reflected with respect to the x axis (that is, inverted) and then scaled as described because the optimum for actin flow is a minimum value rather than a maximum. The scaled data sets were then averaged at each stiffness and again scaled to span from zero to one to create the composite data. To create that composite data fit curves, a logarithmic Gaussian curve was fit to the data without constraining the variance. For the ECFNs, only the actin flow rate and the traction strain energy were used to calculate the composite metric because the other types of data were not available from our previous study (Chan and Odde^7^).

### Statistical analysis

For determining confidence in maxima and minima, the Kruskal–Wallis one-way analysis of variance^30^ was performed to determine if any of the sampled conditions originated from a different distribution than the others. This test was chosen because the sample sizes varied, and the expected shape of the sampled distributions was unknown a priori. The Kruskal–Wallis analysis allows for differing sample sizes and makes no assumption as to the shape of the originating distribution. A subsequent Dunn's test^31^ for multiple comparisons was performed to determine which sample sets, if any, originated from differing distributions than other sample sets. The same analysis was done for comparing the no drug, blebbistatin, cyclo(RGDfV) and both drug cases. For figures comparing only two data sets, the Kruskal–Wallis analysis was performed without Dunn's test because there was no need to correct for multiple comparisons.

The null hypothesis that the stiffness optima for the composite data fit curves are not different from each other was tested using a bootstrapping method. All data points at a particular stiffness from either the untreated or combined drug-treated measurements were shuffled together and a set of measurements were randomly assigned to the untreated case and the remaining measurements were assigned to the combined drug-treated case. The number of measurements assigned to each case was determined from the original data while randomly switching the number of measurements in every shuffle to account to the unequal number of measurements in each case. This was done for random motility coefficient, cell area, aspect ratio, actin retrograde flow rate and traction strain energy for U251 glioma cells. New composite metrics were then calculated for each case, and logarithmic Gaussian curves were fit to the composite data. The logarithmic distance between the maxima for the two fit curves was recorded. This process was repeated 10,000 times to generate a probability distribution of potential stiffness optima differences according to the null hypothesis that there is no difference between the stiffness optima for the two sets of composite data. The experimental stiffness optima difference was then compared with the distribution of differences to determine the confidence to which the two fit optima were significantly different. The same process was performed to test the null hypothesis that the composite data fit curve optima for simulations with low and high motor and clutches are not different from each other.

### Code availability

All codes will be available on our laboratory website (oddelab.umn.edu) or on request from the corresponding author.

### Data availability

The microarray data set generated and analysed in this study from U251 cells cultured on different stiffnesses are available in the National Center for Biotechnology Information Gene Expression Omnibus (GEO) repository (https://www.ncbi.nlm.nih.gov/geo) under accession code GSE95680.

## Additional information

**How to cite this article:** Bangasser, B. L. *et al*. Shifting the optimal stiffness for cell migration. *Nat. Commun.* **8,** 15313 doi: 10.1038/ncomms15313 (2017).

**Publisher's note:** Springer Nature remains neutral with regard to jurisdictional claims in published maps and institutional affiliations.

## Supplementary Material

Supplementary Information Supplementary Tables, Supplementary Figures and Supplementary References

Supplementary Movie 1 Cell migration simulation on a 0.1, 10, and 1000 pN nm-1 substrate using the low motor and clutch parameter set. The simulated cell shape appears in white with the cell trajectory in red. Each motor-clutch module is indicated by a line colored according to the force on that module. The cell body is indicated by the dot at the center of cell which is also colored according to the force on it. Frames were recorded at 15 minute intervals for 20 hours.

Supplementary Movie 2 Untreated U251 glioma cell migration on 4.6, 100, and 200 kPa. Time lapse images of U251 glioma cells on polyacrylamide gel were taken at 15 minute intervals for 10 hours. The cell trajectories were made using our computational cell tracking algorithm.

Supplementary Movie 3 Fluorescent F-actin flow. A fluorescence movie of an EGFP-actin U251 glioma cell on an 9 kPa polyacrylamide gel demonstrates actin retrograde flow at the cell edges. Frames were taken every 2 seconds for 3 minutes.

Supplementary Mov2ie 4 Phase contrast F-actin flow. A phase contrast movie of a U251 glioma cell on a 4.6 kPa polyacrylamide gel demonstrates that the actin flow near the edge of the cell can be visualized without using fluorescent actin. Frames were taken every 2 seconds for 3 minutes.
