# Supplementary material for: Shifting the optimal stiffness for cell migration
Source: Nat Commun. 2017 May 22;8:15313. doi: 10.1038/ncomms15313 (PMC5458120; doi:10.1038/ncomms15313)
Supplement: Supplementary Information — Supplementary Tables, Supplementary Figures and Supplementary References [file ncomms15313-s1.pdf]

Supplementary Table 1: Motor-clutch gene mRNA expression

Myosin Motors

Symbol	Description	Expression	Rank
MYL6	myosin, light chain 6, alkali, smooth muscle and non-muscle	11903	79
MYH9	myosin, heavy chain IIA, non-muscle	5287	318
MYO1B	myosin IB	409	4498
MYO9B	myosin IXB	342	5243
MYH10	myosin, heavy chain IIB, non-muscle	302	5808
MYO1C	myosin IC	235	7067
MYO6	myosin VI	217	7519
MYO5A	myosin VA (heavy chain 12, myoxin)	197	8147
MYL5	myosin, light chain 5, regulatory	153	10116
MYH14*	myosin, heavy chain IIC, non-muscle	109	22699

Actin and Actin Binding Proteins

Symbol	Description	Expression	Rank
ACTB	actin, beta	18685	4
ACTG1	actin, gamma 1	14313	46
PFN1	profilin 1	11265	90
EZR	ezrin	6736	227
CFL1	cofilin 1 (non-muscle)	4192	422
MSN	moesin	4051	437
ACTN1	actinin, alpha 1	2864	670
ARPC3	actin related protein 2/3 complex, subunit 3, 21kDa	2758	706
ARPC5	actin related protein 2/3 complex, subunit 5, 16kDa	2605	753
ARPC1A	actin related protein 2/3 complex, subunit 1A, 41kDa	1935	1033
ARPC2	actin related protein 2/3 complex, subunit 2, 34kDa	1714	1189
PFN2	profilin 2	1537	1309
CAPZA2	capping protein (actin filament) muscle Z-line, alpha 2	1264	1575
FLNB	filamin B, beta (actin binding protein 278)	1126	1766
RDX	radixin	894	2208
ZYX	zyxin	786	2485
CAPZB*	capping protein (actin filament) muscle Z-line, beta	723	2677
ARPC4	actin related protein 2/3 complex, subunit 4, 20kDa	674	2872
CTTN	cortactin	469	3994
ARPC1B	actin related protein 2/3 complex, subunit 1B, 41kDa	455	4096
FLNA	filamin A, alpha (actin binding protein 280)	323	5484
PARVA	parvin, alpha	274	6266
VASP	vasodilator-stimulated phosphoprotein	192	8314
CAPZA1	capping protein (actin filament) muscle Z-line, alpha 1	142	10854
FMN1	formin 1	106	25913
VIL1	villin 1	98	34014

Adhesion Molecules

Symbol	Description	Expression	Rank
CD44	CD44 molecule (Indian blood group)	6359	245
CDH2	cadherin 2, type 1, N-cadherin (neuronal)	3268	575
CTNNA1	catenin (cadherin-associated protein), alpha 1, 102kDa	2737	714
VCL	vinculin	1887	1064
ITGAV	integrin, alpha V (vitronectin receptor, alpha polypeptide, antigen CD51)	1621	1248
ITGB1	integrin, beta 1 (fibronectin receptor, beta polypeptide, antigen CD29 includes MDF2, MSK12)	1391	1441
ITGA3	integrin, alpha 3 (antigen CD49C, alpha 3 subunit of VLA-3 receptor)	1060	1865
CDH11	cadherin 11, type 2, OB-cadherin (osteoblast)	1043	1895
ITGB5	integrin, beta 5	899	2194
TLN1	talin 1	571	3340
ITGB4	integrin, beta 4	552	3464
ITGA5	integrin, alpha 5 (fibronectin receptor, alpha polypeptide)	371	4894
ITGA2	integrin, alpha 2 (CD49B, alpha 2 subunit of VLA-2 receptor)	363	4985
NRCAM	neuronal cell adhesion molecule	346	5179
PXN	paxillin	256	6608
TLN2	talin 2	250	6726
ITGA4	integrin, alpha 4 (antigen CD49D, alpha 4 subunit of VLA-4 receptor)	247	6784
NCAM1	neural cell adhesion molecule 1	194	8240
ITGA6	integrin, alpha 6	145	10680
ITGB3	integrin, beta 3 (platelet glycoprotein IIIa, antigen CD61)	122	14001

*Gene does not appear in Simpson *et al.*[1] list of cell migration genes

Supplementary Table 2: Cell migration simulator parameter values

Symbol	Parameter	Value
N_m	Total number of motors	1,000; 10,000
N_c	Total number of clutches	750; 7,500
A_{tot}	Total possible actin protrusion length	100 μm
v_p*	Maximum actin polymerization velocity	200 nm/s
k_{mod}*	Maximum module birth rate	1 s^{-1}
k_{cap}	Module capping rate	0.001 s^{-1}
l_{in}	Initial module length	5 μm
l_{min}	Minimum module length	0.1 μm
κ_{cell}	Cell spring constant	10,000 pN/nm
$n_{c,cell}$	Number of cell body clutches	10; 100
n_m*	Maximum number of module motors	100; 1,000
F_m	Motor stall force	2 pN
v_m*	Unloaded motor velocity	120 nm/s
n_c*	Maximum number of module clutches	75; 750
k_{on}	Clutch on-rate	1 s^{-1}
k_{off}*	Clutch unloaded off-rate	0.1 s^{-1}
κ_c	Clutch spring constant	0.8 pN/nm
F_b	Characteristic clutch rupture force	2 pN
κ_s	Substrate spring constant	Variable

Supplementary Table 3: Number of experimental observations

		Substrate spring constant (pN/nm)					
		0.01	0.1	1	10	100	1000
Simulation N_m = 1,000 N_c = 750		40	49	35	16	27	12
Simulation N_m = 10,000 N_c = 7,500		24	33	71	34	15	6
	PAG Young's modulus						
	50 Pa	700 Pa	4.6 kPa	9 kPa	20 kPa	100 kPa	200 kPa
U251 motility/area/aspect ratio	15	80	79	47	55	71	66
U251 actin flow		80	138	163	80	138	151
U251 strain energy	9	55	44	61	68	56	
U251 + blebbistatin + cyclo(RGDfV) motility/area/aspect ratio	22	22	126	53	116	117	116
U251 + blebbistatin + cyclo(RGDfV) actin flow		114	189	156	70	64	50
U251 + blebbistatin + cyclo(RGDfV) strain energy	9	68	62	50	41		
U251 + blebbistatin motility/area/aspect ratio			48			37	
U251 + blebbistatin actin flow			68			38	
U251 + blebbistatin strain energy			40	20			
U251 + cylco(RGDfV) motility/area/aspect ratio			58			52	
U251 + cyclo(RGDfV) actin flow			87			134	
U251 + cyclo(RGDfV) strain energy			49	24			

Supplementary Table 4: Significance values for comparisons in Figure 4

Random motility coefficient

	4.6kPa			100 kPa		
	Blebbistatin	cylco(RGDfV)	Both drugs	Blebbistatin	cylco(RGDfV)	Both drugs
No drug	0.36	0.0003	0.0017	0.0004	10^{-11}	0.02
Blebbistatin		0.0002	0.08		0.0003	0.05
cylco(RGDfV)			10^{-8}			10^{-9}

Projected cell area

	4.6 kPa			100 kPa		
	Blebbistatin	cylco(RGDfV)	Both drugs	Blebbistatin	cylco(RGDfV)	Both drugs
No drug	10-8	0.0001	10^{-16}	0.65	10^{-5}	0.0003
Blebbistatin		0.01	0.0004		10^{-5}	0.0007
cylco(RGDfV)			10^{-9}			0.31

Cell aspect ratio

	4.6 kPa			100 kPa		
	Blebbistatin	cylco(RGDfV)	Both drugs	Blebbistatin	cylco(RGDfV)	Both drugs
No drug	10^{-8}	0.06	10^{-9}	0.63	0.24	0.9
Blebbistatin		10^{-4}	0.23		0.16	0.6
cylco(RGDfV)			0.0003			0.22

Actin flow rate

	4.6 kPa			100 kPa		
	Blebbistatin	cylco(RGDfV)	Both drugs	Blebbistatin	cylco(RGDfV)	Both drugs
No drug	10^{-6}	0.01	10^{-13}	0.08	10^{-12}	0.17
Blebbistatin		10^{-9}	0.4		10^{-8}	0.014
cylco(RGDfV)			10^{-15}			10^{-5}

Traction strain energy

	4.6 kPa			9 kPa		
	Blebbistatin	cylco(RGDfV)	Both drugs	Blebbistatin	cylco(RGDfV)	Both drugs
No drug	10^{-11}	10^{-11}	10^{-8}	10^{-8}	10^{-6}	10^{-10}
Blebbistatin		0.9	10^{-4}		0.0054	0.008
cylco(RGDfV)			10^{-5}			0.33

A

	Measured Young's Modulus						
	50 Pa	740 Pa	4.6 kPa	9.3kPa	19.8kPa	98.5kPa	195 kPa
40% Acrylamide (μL)	75	75	100	125	250	500	500
2% Bis-acrylamide (μL)	20	50	100	50	50	250	450
1 M HEPES (μL)	10	10	10	10	10	10	10
FluoSpheres (μL)	10	10	10	10	10	10	10
Deionized Water (μL)	885	855	780	805	680	230	30
APS (μL)	6	6	6	6	6	6	6
TEMED (μL)	4	4	4	4	4	4	4

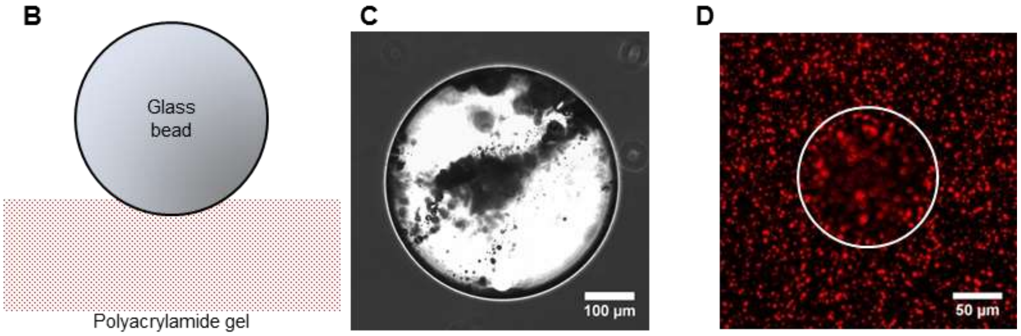

Supplementary Figure 1: Formulations and stiffness measurement of polyacrylamide gels. (A) Recipes for each stiffness of polyacrylamide gel. (B) Schematic of a glass bead indenting a polyacrylamide gel containing crimson fluorospheres. (C) 470 μm diameter glass bead used for stiffness measurement. (D) Indentation in a 0.7 kPa polyacrylamide gel caused by a 470 μm diameter glass bead. The indented region is out of focus compared to the surrounding gel.

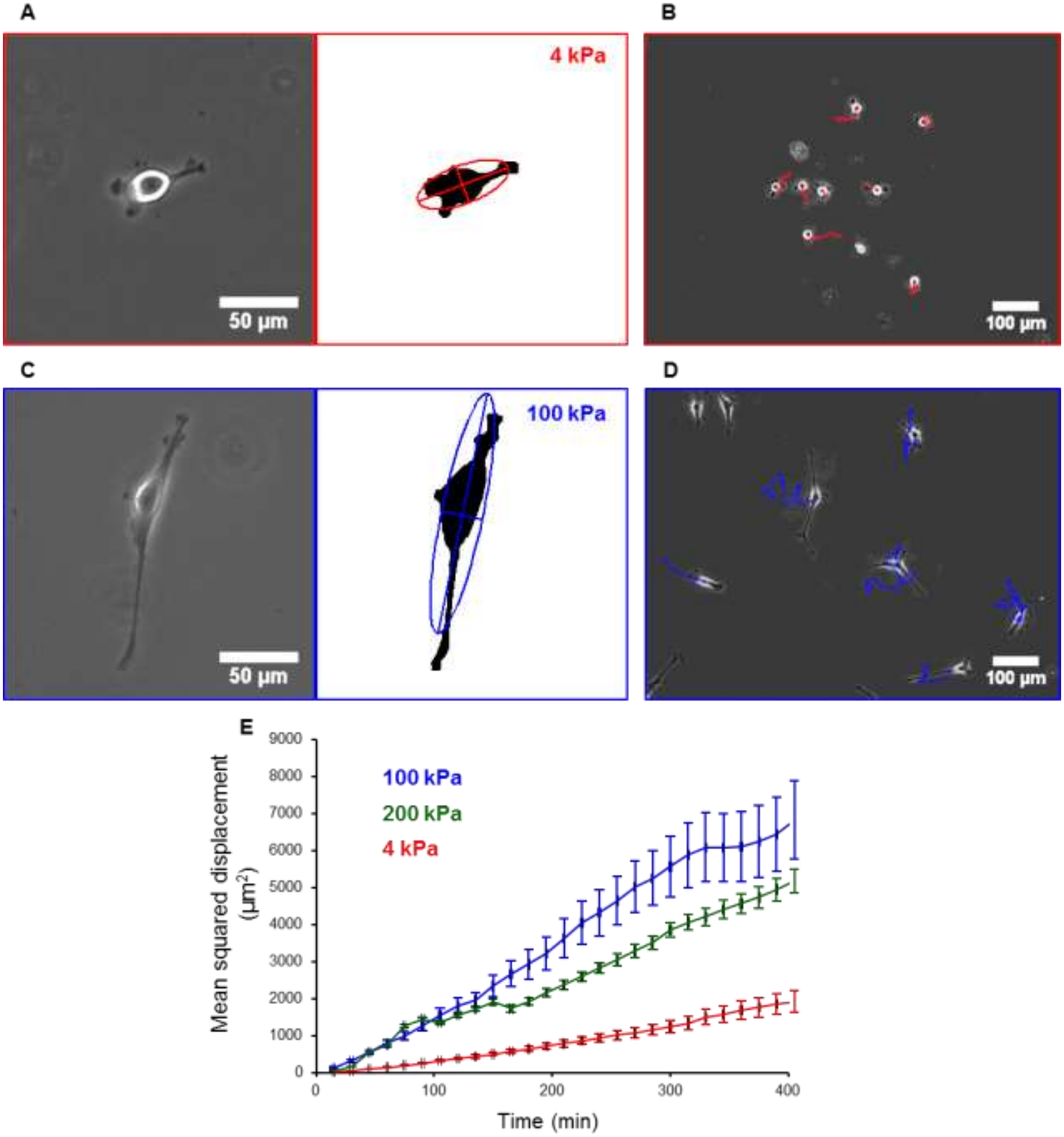

Supplementary Figure 2: Analysis of U251 glioma cell morphology and migration. (A) A U251 glioma cell on a 4.6 kPa polyacrylamide gel with its corresponding image segmentation and fitted ellipse. (B) Trajectories of U251 glioma cells on 4.6 kPa over 15 hours. (C) A U251 glioma cell on a 100 kPa polyacrylamide gel with its corresponding image segmentation and fitted ellipse. (D) Trajectories of U251 glioma cells on 100 kPa over 15 hours. (E) Averaged mean squared displacement versus time plots for U251 glioma cell migration on 4.6 kPa, 100 kPa, and 200 kPa polyacrylamide gels. All error bars are s.e.m.

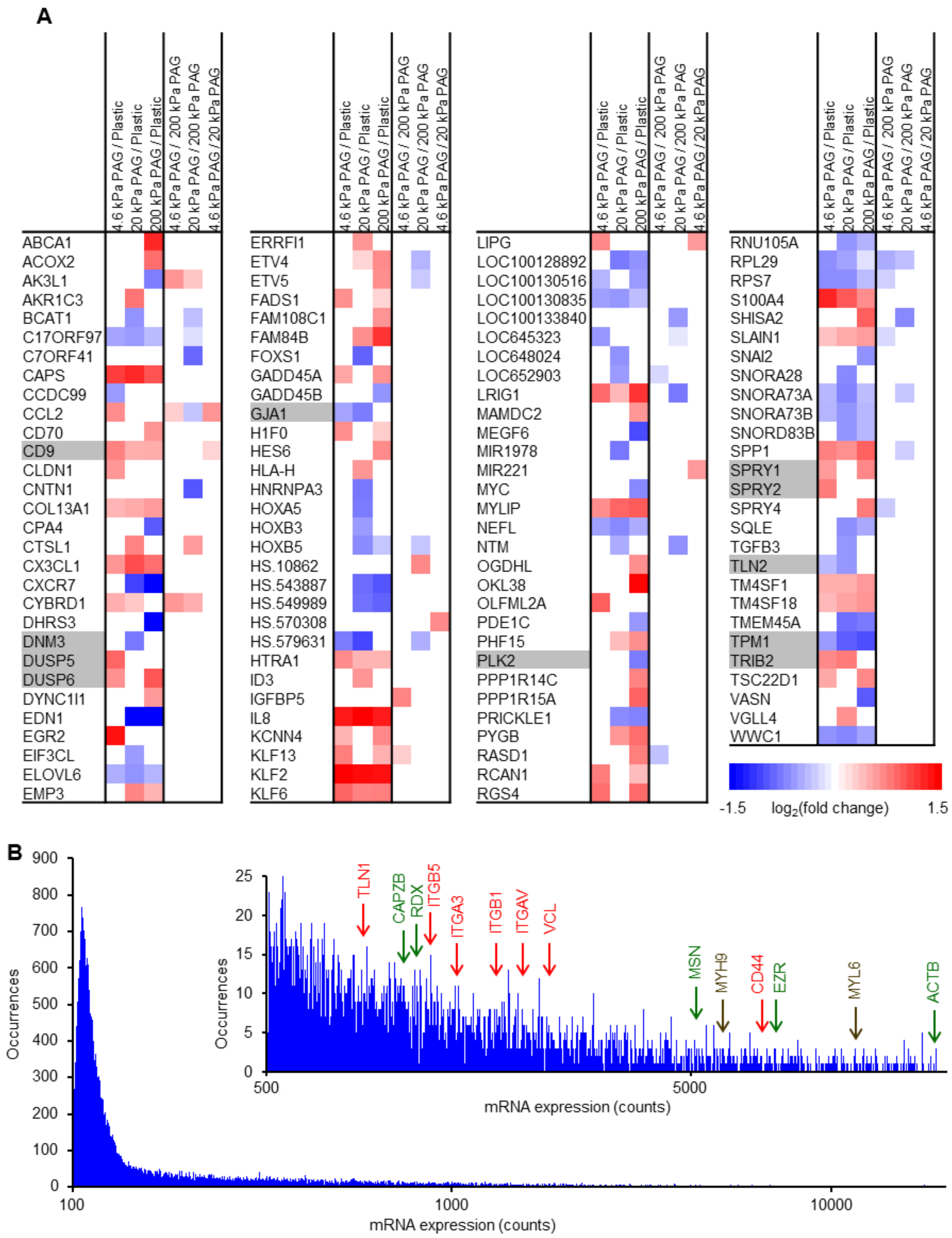

Supplementary Figure 3: U251 glioma cell gene mRNA expression. (A) List of genes that had at least a 1.5 fold-change in mRNA expression with $p<0.01$ for any of the six comparisons

among substrate conditions. Genes with significantly different comparisons are color coded according to fold-change in expression. Genes which appear in the Simpson *et al*.[1] list of cell migration genes are highlighted in grey. (B) Histogram of mRNA expression for all genes measured with selected cell migration genes identified.

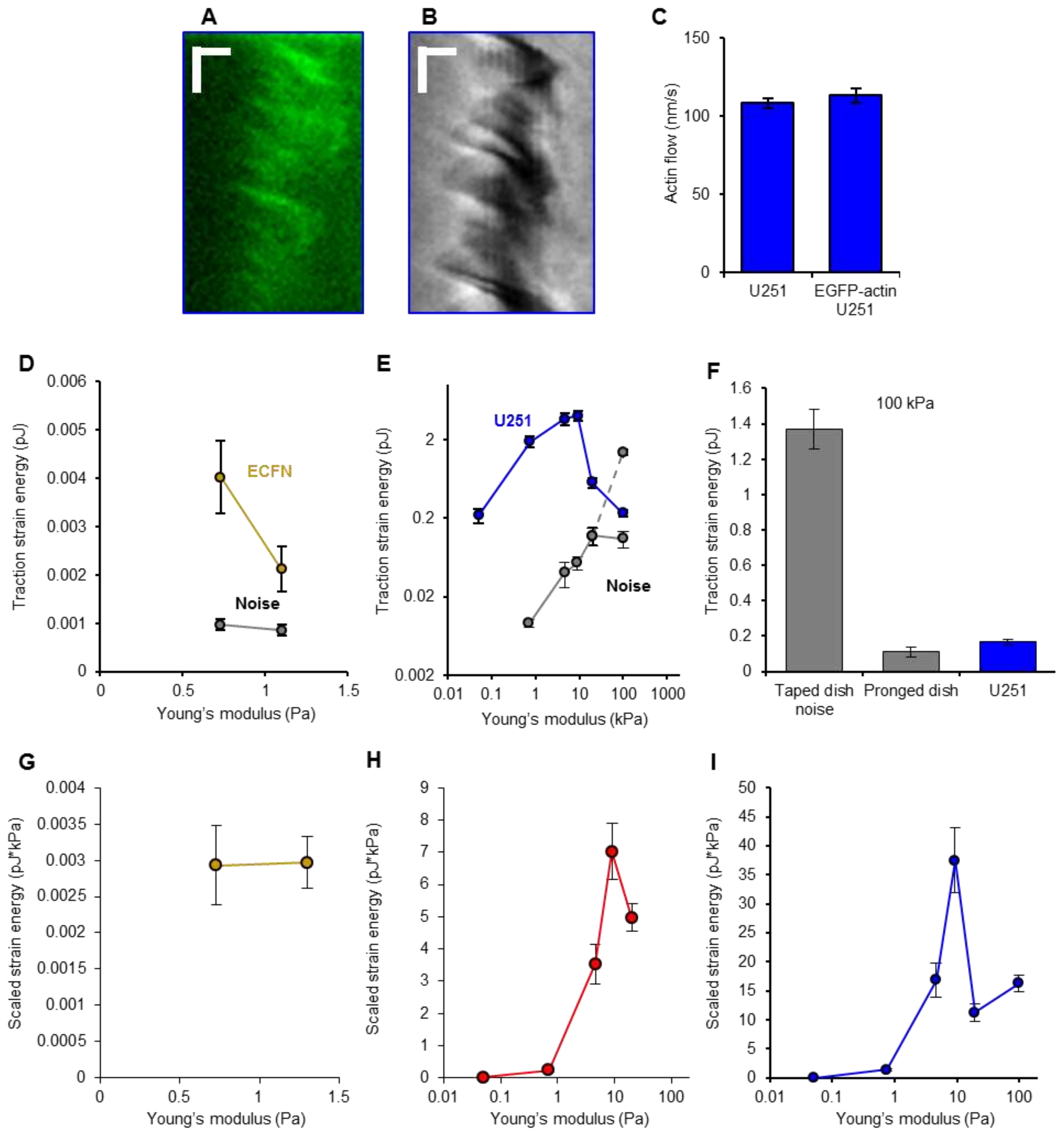

Supplementary Figure 4: Actin flow and traction strain energy validation. (A) Fluorescence kymograph of actin flow in an EGFP-actin U251 glioma cell on a 4.6 kPa polyacrylamide gel. Horizontal bar is 2 μm. Vertical bar is 30 s. (B) Phase contrast kymograph of actin flow in a U251 glioma cell on a 4.6 kPa polyacrylamide gel. Horizontal bar is 2 μm. Vertical bar is 30 s. (C) On a 4.6 kPa polyacrylamide gel, actin flow in EGFP-actin U251 glioma cells is not

significantly different from actin flow in U251 glioma cells ($p=0.7$). (D) ECFN strain energy is above the noise floor of the measurement. (E) U251 glioma cell strain energy is above the noise floor except for the measurement on 100 kPa. For this stiffness, a new pronged microscope stage insert was used to better secure the dish during the experiment. (F) The U251 glioma cell traction strain energy on 100 kPa is greater than the noise floor using the pronged stage ($p=0.009$). (G) Scaled strain energy for ECFNs. The scaled strain energies are not significantly different ($p=0.9$). (H) Scaled strain energy for U251 glioma cells with 6 μM blebbistatin and 0.6 μM cylco(RGDfV). The correction eliminates the significant difference between 9 kPa and 20 kPa ($p=0.3$). (I) Scaled strain energy for U251 glioma cells. The maximum occurs between 4.6 kPa-100 kPa ($p=0.09$). All error bars are s.e.m.

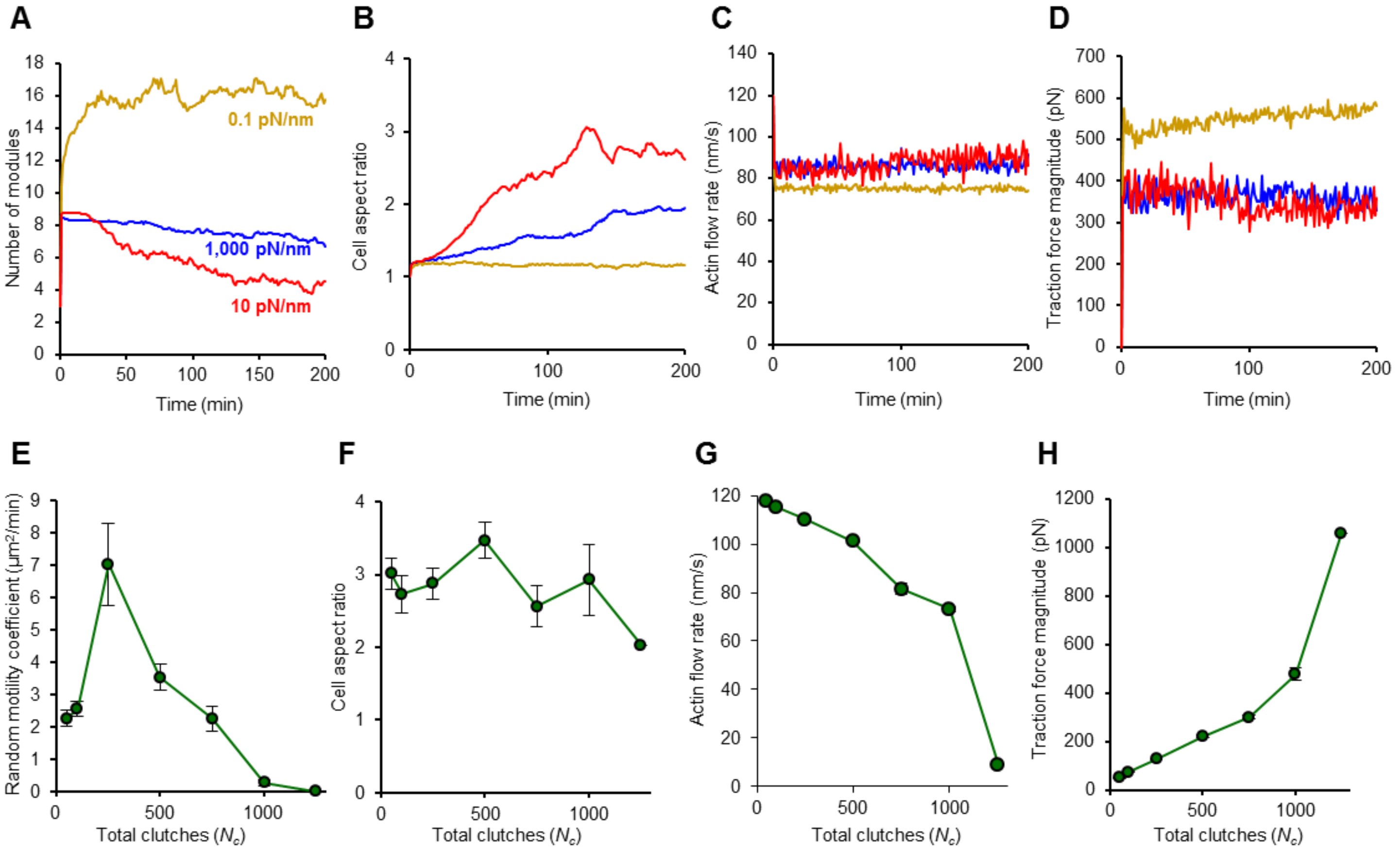

Supplementary Figure 5: Steady state analysis and adhesivity simulations. (A-D) Time course data for the low motor and clutch parameter set shows that steady state is reached after about 100 minutes on 0.1 pN nm^{-1}, 10 pN nm^{-1}, and 1,000 pN nm^{-1} substrates for number of motor-clutch modules (A), cell aspect ratio (B), actin retrograde flow rate (C), and traction force magnitude (D). Each type of data was recorded at one minute intervals and averaged over 40, 34, and 15 simulations for 0.1 pN nm^{-1}, 10 pN nm^{-1}, and 1,000 pN nm^{-1} substrates, respectively. (E-H) Adhesivity results obtained from the cell migration simulator. Each simulation was run on a 10 pN/nm substrate with 1,000 total motors (N_m) and 100 maximum motors per modules (n_m*). The total clutches (N_c) were varied while maintaining the maximum number of clutches per module (n_c*) at 1/10 of the total clutches. (E) Random motility coefficient is maximal between 100-750 total clutches (p=0.02). (F) Cell aspect ratio does not significantly change (p=0.3). (G) Actin flow rate decreases with total clutches. (H) Traction force magnitude increases with total clutches. All error bars are s.e.m.

Supplementary References

1. Simpson, K. J. *et al.* Identification of genes that regulate epithelial cell migration using an siRNA screening approach. *Nat. Cell Biol.* **10,** 1027–38 (2008).
